# A hypothesis study on a four-period prevention model for high altitude disease

**DOI:** 10.1186/s40779-018-0150-0

**Published:** 2018-01-24

**Authors:** Xian-Sheng Liu, Xiang-Rong Yang, Lu Liu, Xian-Kui Qin, Yu-Qi Gao

**Affiliations:** 1 Institute of Medicine and Hygienic Equipment for High Altitude Region, College of High Altitude Military Medicine, Third Military Medical University, No. 30 Gaotanyan Street, Shapingba District, Chongqing, 400038 China; 2 Department of Medical Affairs, The 5th Hospital of the Chinese PLA, No. 893 Shengli Street, Xingqing District, Yinchuan, Ningxia 750003 China; 3 Key Laboratory of High Altitude Environmental Medicine, Third Military Medical University, Ministry of Education, No. 30 Gaotanyan Street, Shapingba District, Chongqing, 400038 China; 4 Key Laboratory of High Altitude Medicine of PLA, No. 30 Gaotanyan Street, Shapingba District, Chongqing, 400038 China; 5 Department of Health Service, Training Base of Health Service, Third Military Medical University, No. 30 Gaotanyan Street, Shapingba District, Chongqing, 400038 China; 6 Department of Outpatient, PLA No.96819 Unit, No. 2 Heping Road, Chaoyang District, Beijing, 100085 China

**Keywords:** High altitude disease, High altitude health service, Prevention model

## Abstract

**Background:**

High altitude disease (HAD) can reduce combat effectiveness and damage the health of soldiers at high altitudes. The objective of this hypothesis study is to build a four-period prevention model for high altitude disease that can be applied at high altitudes of over 3000 m.

**Presentation of the hypothesis:**

We divided the time at high altitude into nine periods, with three stages from the ascent preparation to the descent to the plain, and applied a continuous dynamic and systematic four-period prevention model across the nine periods. Each period of three stages has its own different measures and targets high altitude health care services for the prevention of high altitude disease. A standard four-period prevention model for high altitude disease was constructed for the high altitude health services at the population level.

**Testing the hypothesis:**

Our hypothesized HAD prevention model represents a continuous dynamic and systematic four-period prevention model across the nine periods. This hypothesis can be tested from three aspects. The first one isassessment of soldiers' operating efficacies. The second is comparison of the long-term high altitude population health basic data and development and utilization of big data. The third is descent population health status comparative study and historical retrospective study on prevention.

**Implications:**

As we know, it is necessary to protect soldiers’ health through the ascent and descent. Through the standard four-period model, we can protect soldiers’ health by preventing high altitude diseases, screening the susceptible population, securely tracking their location and maintaining soldiers’ health statuses; we also maintain their operational capabilities, eliminate their psychological fears and ease their family troubles.

## Background

High altitude disease (HAD) is the most common sickness in areas with altitudes over 3000 m. China has the world’s largest highland, with a population of 60 million native and immigrant highlanders, and the world’s longest highland border. Highland areas at altitudes over 3000 m comprise about one-fourth of China. With the development of China’s economy, the housing, transportation, meals, and medical conditions for soldiers have been largely improved. However, HAD is the main reason for reduced combat effectiveness and health damage of soldiers at high altitudes and influences the results of war on the highland plateau. Berger et al. [1] found that the heart was protected when preconditioning was performed in a different organ than the target, which is called remote ischemic preconditioning (RIPC). These pathways are also thought to play a role in high altitude disea**s**es. Li et al. [2] has proved that Gao Yuan Kang capsules have remarkable anti-oxidation and anti-hypoxia effects at high altitudes, and they can effectively prevent the incidence of acute high altitude diseases (AHAD). Altitude illness affects 25% to 85% of travelers to high altitudes, depending on their rate of ascent, home altitude, individual susceptibility, and other risk factors. Descent is mandatory for all persons with high altitude cerebral or pulmonary edema. The most accepted method of preventing acute mountain sickness and high altitude cerebral edema is to ascend slowly [3]. In this paper, “soldiers” refers to Han Chinese lowland soldiers. A population-level prevention campaign to combat HAD may become an important focus of our research in the future.

**Fig. 1:**
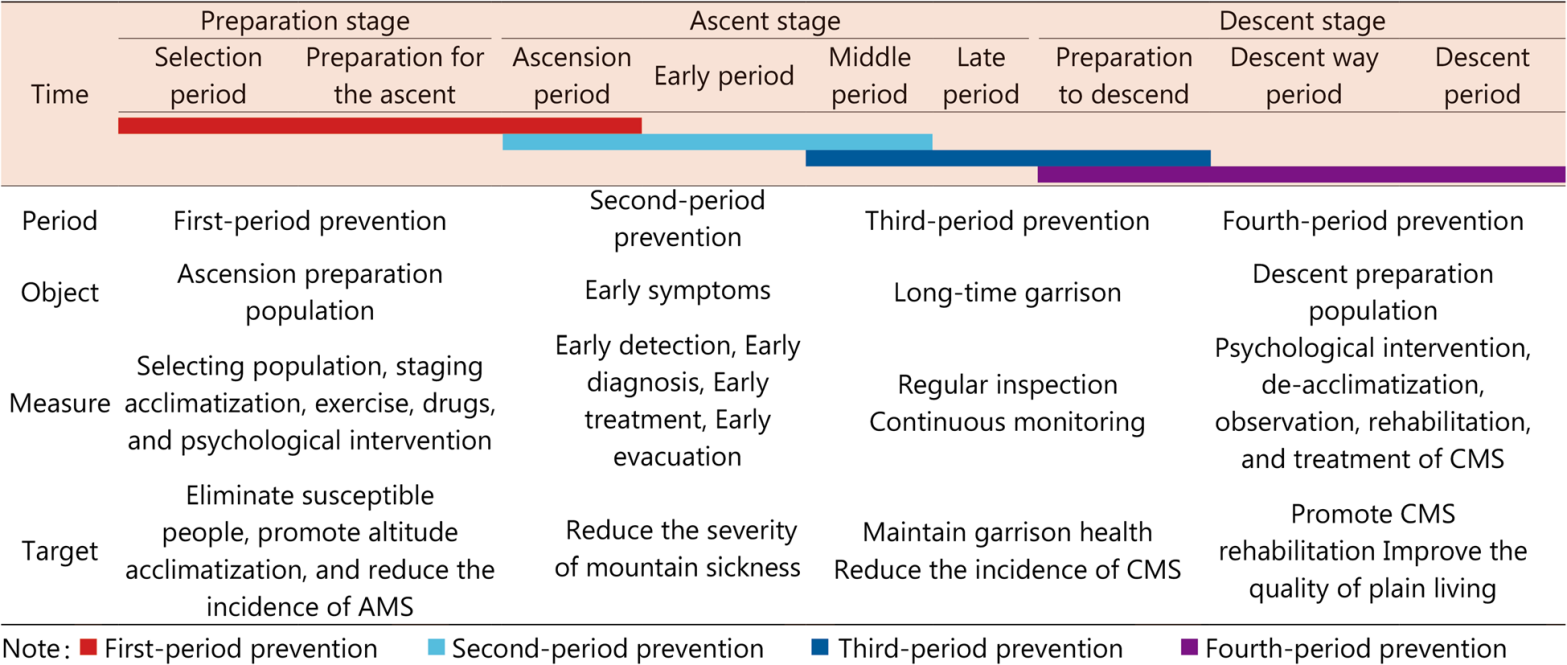
Four-period prevention model for mountain sickness

At present, the main form of prevention from HAD is short-term, emergency prevention, including staging acclimatization, adaptive training, drugs and rich oxygen. There is a lack of effective screening methods, and there is no series of protection system strategy. The most intensive prevention is implemented before entering the plateau or being stationed at the plateau. We have not tracked the health of soldiers’ descent nor established health data for soldiers. Therefore, we divided the time spent at high altitudes into nine periods, with three stages from the ascent preparation to the descent to the plain, and applied the four-period continuous dynamic and systematic prevention model across the nine periods. The main object, target and appropriate prevention measures in each period are described (shown in Fig. 1).

## Presentation of the hypothesis

HAD is common on the plateau and is unique to high altitude areas. It is a system of clinical symptoms caused by changes in pathophysiology as well as body disorders that occur after ascending to an hypoxic environment with an altitude over 3000 m. We need a continuous protection program to prevent HAD and maintain soldiers’ health. The targets of this prevention program are Han Chinese soldiers from the plains. We divided the time at altitude into nine periods, with three stages from the preparation for ascent to a high altitude to the time after soldiers descend to a low altitude (Fig. 1). The three stages are the preparation stage, the ascent stage and the descent stage. The preparation stage includes the selection period and preparation for the ascent. The ascent stage consists of the ascension period, an early period, a middle period and a late period. The descent stage includes the preparation to descend, a descent way period and a descent period. Using the nine periods, we apply a four-period prevention model.

### The first period for HAD prevention

The first period of prevention includes the preparation stage and the ascension period of the ascent stage. The concrete measures taken at this stage include personnel screening, staging acclimatization, exercise, drugs, and psychological intervention.

#### Personnel screening

Compared to other diseases, HAD is only experienced regionally. If you do not enter the plateau’s hypoxic environment, you will not get HAD. According to previous research, the individual response to hypoxia is different. Some of the population is more susceptible to HAD than others. Therefore, the first thing we can do is to screen the population of soldiers for those who are less susceptible to HAD to prepare to ascend to high altitudes. In this way, we can reduce the incidence of HAD from the beginning. Many factors are related to of the potential to experience HAD, such as resident altitude, health, age, smoking, and so on [4]. However, all of these indicators are non-quantitative, and we need quantitative indicators. Recently, some genes have been found to be related to HAD, and they may become our research focus in the future [5].

#### Staging acclimatization

Staging acclimatization is an internationally used method for reducing acute mountain sickness (AMS). The main procedure divides the ascent into three steps at low, middle, and high plateaus [6]. A standardized method and steps for staging acclimatization were published by the U.S. Army Research Institute of Environmental Medicine in the *Altitude Acclimatization Guide* [7].

Under certain special conditions, the army cannot perform staging acclimatization according to the guide. To supplement, people can exercise appropriately in the plain or at an altitude of 2000–2500 m. It is more effective to strengthen staging acclimatization with climbing, running, and swimming; alternatively, exchange of garrisons on the plain and middle plateau can be effective. Previous research has shown that short-time hypoxia with physical activity can significantly reduce the incidence of AMS at a simulated altitude of 4200 m. At the same time, it can also reduce the severity of AMS and improve soldiers’ work abilities [8]. In addition, using low-pressure tank hypoxia repeatedly for one-half to 1 h periods every 1–3 days can produce similar results as exposure to a high altitude hypoxic environment, resulting in acclimatization [9]. Currently, this small, convenient machine includes equipment such as a low-oxygen respirator [10].

#### Drugs

Some time is needed to become acclimatized after staging an acclimatization exercise. However, in the case of a special emergency, the army cannot wait for the soldiers to become acclimatized. Drugs can strengthen the body in preparation for the hypoxic environment in a short period of time. Practice has proven that drugs that can improve the body’s hypoxia endurance are good for an accelerated protection effect in the rapid deployment of soldiers to the plateau. In China, these drugs can be divided into two types: traditional Chinese herbal medicine and Western medicine. 1) Chinese herbal medicine: Currently, research in traditional Chinese medicine considers HAD to be a result of Qi deficiency, Blood deficiency, and Yin injury. Therefore, the body’s hypoxia endurance can be improved by tonifying Qi-Blood-Yin. Research in China on the use of drugs that increase the body’s hypoxic endurance for HAD prevention has focused on Chinese herbal medicine, such as the *Codonopsis pilosula*, *Rhodiola rosea*, milkvetch root–*Poria cocos* compound, *Dracocephalum heterophyllum*, *Dracocephalum tanguticum* Maxim, Astragalus, *Cordyceps sinensis*, Acanthopanax, and *Rosa acicularis* [11–14]. 2) Western medicine: Acetazolamide and dexamethasone are commonly used drugs. Nifedipine is the first drug to prevent high altitude pulmonary edema (HAPE) [15, 16]. For most people, these drugs can improve the efficiency of gas exchange and movement, ease the acute symptoms of HAD, improve sleep, and maintain oxygen saturation. If combined with aspirin, these drugs can also improve oxygen content in tissue, reduce prostaglandin synthesis, and be effective in the treatment of plateau headache. In addition, *Ginkgo biloba* extract (EGb761) and theophylline are efficient in preventing AMS and promoting acclimatization to the plateau altitude [17].

#### Psychology

Psychology is one of the most important influences on AMS. Overcoming the fear of the plateau altitude can also have a good preventive effect for AMS. In 1979, Olive et al. [18] proved that psychology has a deep relationship with AMS. Oliver et al. [18] also proved that a population with bad psychology experiences a high incidence of AMS. The appropriate time for psychological intervention is 1 week before ascent to a high altitude [19]. Health education and instruction about the plateau environment can eliminate worries, fear, or depression in new soldiers serving in Tibet and reduce the incidence of AMS.

### The second period of HAD prevention

The second period of prevention includes three periods: the ascension period and the early and middle periods of the ascent stage. The main method of preparation in the second period is observation of early HAD symptoms in the population new to high altitudes. Our practice follows the four “early” actions, including early detection, early diagnosis, early treatment, and early evacuation. The purpose of second period preparation is to reduce the incidence and severity of AMS, reduce the severity of HAPE or high altitude cerebral edema (HACE), and maintain the operational ability of soldiers who have experienced rapid ascension to high altitudes [20]. The “static” method is widely used, which creates a quiet living environment, controls physical activity, and promotes the body’s ability to compensate for and adapt slowly to the hypoxic environment [21]. Practical methods during this period, such as keeping warm, limiting alcohol, controlling water intake, and maintaining a diet high in carbohydrates, can also enhance the body’s resistance to the hypoxic environment [22, 23]. Currently, there are two ways to grade AMS symptoms in China. One is China’s National Military Standard: the principles of diagnosis and treatment of AMS (GJB1098–91). Another is the internationally recognized Lake Louise AMS Scoring System.

### The third period of HAD prevention

After being in high altitude areas over 3000 m for 6 months, we enter the stage of the third period of prevention, which includes the middle and late periods of the ascent stage and the preparation to descend of the descent stage. By now, most of the soldiers have adapted to the hypoxic environment and have become completely acclimated [24]. The central nervous, circulatory, respiratory, digestive, blood, reproductive, and endocrine systems have changed. Part of the population gradually develops chronic mountain sickness (CMS). CMS is a common high altitude disease with the main pathological characteristics of excessive proliferation of red blood cells, pulmonary hypertension and hypoxemia [25]. If CMS cannot be treated in a timely manner, severe cases can lead to serious heart disease, heart failure, and even loss of life. The main methods of preparation during the third period are regular inspections and continuous tracking of health status, timely diagnosis, and CMS treatment. Currently, the main CMS threats to health among the garrison are high altitude polycythemia, high altitude heart disease, and HAD with abnormal blood pressure.

### The fourth period of HAD prevention

The fourth period of prevention applies to the population preparing to leave a high altitude region. Fourth period includes the preparation to descend, the descent way period, and the descent period. The fourth period prevention measures are different for each soldier. Generally, they include two aspects: one is to promote the soldier’s absolute de-acclimatization, and the other is to provide systematic observation, treatment, and rehabilitation to CMS patients.

#### De-acclimatization

After the population has adapted to the hypoxic environment for a certain time, descending rapidly to the plain can produce symptoms that are similar to AMS, which is called “de-acclimatization” or “low-altitude reaction.” The symptoms include weakness, drowsiness, dizziness, chest tightness, and diarrhea. If we ignore these symptoms, they may lead to serious damage to one’s health. Therefore, we include a fourth period of prevention in our study. The first task during the fourth period is health education in the preparation period of the descent stages. Through this education, we can make this group recognize the importance of the preparatory work to de-acclimatize before they descend to the plain. The group should not think that everything will be normal after descending to the plain. The second task during the fourth period is to promote the de-acclimatization of the descending population. Previous studies have shown that the time for de-acclimatization differs between individuals in a range from 3 months to 3 years [26]. The severity of symptoms is significantly related to age, time at high altitudes, and the altitude of the garrison.

#### CMS rehabilitation treatment

There are currently no satisfactory methods to treat CMS. The common methods to treat CMS include support therapies such as improving the oxygen supply and reducing oxygen consumption, reducing the number of erythrocytes, and improving microcirculation. The CMS rehabilitation treatment is related to the daily life of the population after soldiers descend to the plains and undergo acclimatization. The greatest difficulty with treatment is how to continuously monitor the soldiers’ health.

## Testing the hypothesis

In the past, prevention of HAD was more focused on the ascension period and lacked whole health prevention, especially in the descent population. Our hypothesized HAD prevention model represents a continuous dynamic and systematic four-period prevention model across the nine periods. These nine periods can form a closed loop from ascent time to descent time.

The hypothesized new model is a summary of past research, as well as a guide or goal for future research. The argument, testing, creation and implementation of this model are a long-term process involving many aspects, such as medicine, military affairs, geography, policies and regulations. This hypothesis can be tested from three aspects. The first one is assessment of soldiers’ operating efficacies, including the incidence of HAD, severity of HAD, fine work ability, and brain function. The second is comparison of the long-term high altitude population health basic data and development and utilization of big data. The third is descent population health status comparative study and historical retrospective study on prevention.

## Implications of the hypothesis

HAD is a common and frequently occurring disease in soldiers serving on the plateau. Previous literature has reported the incidence of HAD as being 30% to 90% of the population if there is no intervention. HAD affects soldiers’ operational capabilities and causes some psychological burdens for the soldiers who are prepared to ascend to high altitudes as well as for their families. Therefore, we built the four-period prevention model for HAD to use different methods of intervention at different times and identified the main purpose for each period. There are four aspects underlying the main purpose for a four-period HAD prevention plan: 1) Reducing pressure on high altitude medical support services. 2) Improving the health of soldiers serving at high altitudes. 3) Eliminating unnecessary fears in soldiers and their families at high altitudes. 4) Maintaining and improving the operational capabilities of soldiers serving at high altitudes.

## Abbreviations

AMS: Acute mountain sickness
CMS: Chronic mountain sickness
GJB: China National Military Standard
HACE: High altitude cerebral edema
HAD: High Altitude Disease
HAHS: High altitude health services
HAPE: High altitude pulmonary edema
